# Marine citizenship: The right to participate in the transformation of the human-ocean relationship for sustainability

**DOI:** 10.1371/journal.pone.0280518

**Published:** 2023-03-13

**Authors:** Pamela M. Buchan, Louisa S. Evans, Margherita Pieraccini, Stewart Barr

**Affiliations:** 1 Department of Geography, Faculty of Environment, Science and Economy, University of Exeter, Exeter, United Kingdom; 2 University of Bristol Law School, Bristol, United Kingdom; National Sun Yat-sen University, TAIWAN

## Abstract

Marine citizenship is a relatively new field of enquiry and research to date has focused on individual pro-environmental behaviour change as an expression of responsibility towards the ocean. The field is underpinned by knowledge-deficit and technocratic approaches to behaviour change such as awareness raising, ocean literacy, and environmental attitudes research. In this paper we develop an interdisciplinary and inclusive conceptualisation of marine citizenship. We use mixed methods to study the views and experiences of active marine citizens in the United Kingdom to broaden understandings of marine citizens’ characterisation of marine citizenship, and their perceptions of its importance in policy- and decision-making. Our study shows that marine citizenship entails more than individual pro-environmental behaviours, and includes public-facing and socially collective political actions. We contextualise the role of knowledge, finding more complexity than normative knowledge-deficit approaches permit. We illustrate the importance of a rights-based framing of marine citizenship which incorporates political and civic rights to participate in the transformation of the human-ocean relationship for sustainability. Recognising this more inclusive approach to marine citizenship, we propose an expanded definition to support further exploration of the multiple dimensions and complexities of marine citizenship and to enhance its benefits for marine policy and management.

## 1. Introduction

The ocean is fundamental to climate regulation and has immense importance to society through sustenance, the economy and ecosystem services [1, 2]. Despite concerted efforts to halt ocean degradation, anthropogenic marine environmental degradation persists through a wide range of impacts such as overfishing [3], marine litter [4, 5], microplastics [6], pollution [7], ocean acidification and warming [8], and global climate change [9]. There is an urgent imperative to improve the human-ocean relationship for ecological and human benefit, as recognised by Sustainable Development Goal 14 [10]. Sustainable management of the ocean as a common good goes beyond regulating extractive uses of the ocean and requires institutions and policy to effectively engage the wider public. Public deliberation, reflective process, and consensus-building are needed to set the value-base for future decision-making on sustainable ocean use and governance [11], especially given the contested and open-ended meaning of sustainability [12].

The urgency of ocean degradation and climate change has led to a range of strategies seeking to alter the way nature is valued and managed by society. This includes approaches aimed at raising awareness and delivering environmental education to promote individual behaviour change [13–16]. Marine citizenship, understood as taking personal responsibility for the oceans, has been identified as a potential policy tool to engage the wider public in marine environmental issues via increased awareness, sense of responsibility, and changed behaviour [17]. Though positive relationships have been found between knowledge and concern [13, 18], and knowledge and some actions, including disposal of marine litter and reduced wildlife disturbance [19, 20], the directionality of the relationship is not determined and there is evidence that even high concern and awareness do not always result in more pro-environmental behaviours [13]. Wider research into public engagement with science indicates there is a value-action gap [21, 22] which means raised knowledge, concern or awareness may still not lead to behaviour change [23]. Indeed studies suggest any effect of knowledge on pro-environmental behaviours is mediated by other factors, for example efficacy and values [24] and even political partisanship [25].

Responding to a call for further interrogation of marine citizenship [17], this paper examines how marine citizenship can be reconceptualised through an inclusive and interdisciplinary lens, which recognises individual responsibility as well as participatory rights. Drawing on Faulks’ [26] notions of ‘thin’ and ‘thick’ citizenship, we challenge interpretations of environmental citizenship both as solely a set of pro- environmental behaviours and as predominantly driven by knowledge and awareness (‘thin’ citizenship) [16], in the context of the marine environment, and instead situate it as a set of individual and collective political acts (‘thick’ citizenship). Through a broader (political and environmental) citizenship lens, particular consideration is given to the right to participate in transforming society’s relationship with the sea, and the nature of this participation as perceived by active marine citizens.

### 1.1 Marine and ocean citizenship

Marine citizenship first appears in a paper by Fletcher & Potts [27] as “ocean citizenship”, where three key concepts are stated: that the ocean is a common good; that individuals, including publics, impact the ocean; and that environmental citizenship relates to the geographies of people as physically situated in the environment. In the early literature, marine citizenship is seen to grow from ocean literacy and is defined as “*having understanding of the individual rights and responsibilities towards the marine environment*, *having an awareness and concern for the marine environment and the impacts of individual and collective behaviour*, *and having a desire to have a role in ensuring on-going sustainable management of the marine environment*.” [28, p294]. Through behaviour changes, promoted by education and awareness raising, marine citizenship is considered a potential means of promoting marine sustainability [29, 30]. To illustrate this educational focus, a literature search for “*marine citizenship*” and “*ocean citizenship*” in ISI Web of Science yields only 16 papers (at completion of the study in March 2021), eleven of which have a focus on education and eight on awareness raising, an emphasis particularly strong in ocean research [31]. Only two consider broader drivers of marine citizenship, such as place attachment, personality variables and socio-economic factors. Despite rights appearing in the marine citizenship definition [29], no research has since been conducted to investigate what the rights are, nor has it considered existing participatory rights in environmental decision-making. This relatively reductionist approach to the concept is likely due to environmental citizenship’s roots in environmental education, which developed with a linear relationship between environmental literacy and environmental action at the heart of its pedagogy [16]. The research on marine citizenship to date has not engaged with political citizenship theory nor conceptualisations of marine and environmental citizens as political, civic-engaged, environmentally responsible people, who are more likely to engage in the desired pro-environmental behaviours [23]. In this paper, viewing marine citizenship through a political lens, we proceed to offer a first characterisation of these as marine participatory rights.

### 1.2 Marine citizenship participatory rights

Marine citizenship participatory rights have not been characterised to date in the literature. Ocean-policy rights have typically considered substantive environmental and conservation rights [31], such as the right to clean water, rather than procedural rights, such as the right to participate in marine decision-making. Using Marshall’s framework of civil (legal), political (participation) and social rights (life) [32], the substantive environmental rights can be considered social rights. Marine citizenship rights however are also political rights–the right to self-govern and participate in the way humans interact with the ocean. Civil rights, stipulated by legislation on procedural participation, are also important in this context. Dobson [33] argues for a breaking down of citizenship dichotomies where being responsible becomes a right. In this vein, we argue that marine citizenship rights therefore are concerned with the right to participate in the transformation of the human-ocean relationship, and that this is in the widest understanding, inclusive of social action, civic participation, and procedural participation. A useful first examination of the rights aspect of marine citizenship is therefore to interrogate marine citizens’ participatory experiences and situate these within the existing procedural environmental rights landscape.

Principle 10 of the 1992 Rio Declaration on Environment and Development [34, p3] put emphasis on the procedural elements of sustainability, stating that “*environmental issues are best handled with participation of all concerned citizens*, *at the relevant level”*. It highlighted the three dimensions of participation, including access to information, participation in environmental decision making, and access to justice. Such elements were given legal backing and transformed into rights at the regional level by the United Nations Economic Commission for Europe (UNECE) Convention on Access to Information, Public Participation in Decision-Making and Access to Justice in Environmental Matters (the Aarhus Convention) in 1998 [35]. The Convention empowers environmental NGOs and gives a voice to the wider public. However, it has also faced criticism in the field of environmental law for ill-defining the nature of participation; reinforcing existing power imbalances through, for example, making a distinction between the public and the public concerned, placing the onus on developers to determine the latter, and the limitations in NGO ability to represent marginalised groups; and for specificity only to environmental NGOs and not, for example, justice or rights focused NGOs [36–41]. In this study, we use this participatory and environmental justice legislative framework to examine marine citizens’ experiences of exercising these rights.

### 1.3 Expanding the conceptualisation of marine citizenship

To summarise the current state of the art, the field of marine citizenship is in its early stages of development and its main limitations are three-fold:

- The analytical focus on private, individual behaviour change (‘thin’ citizenship) at the expense of more public or collective citizenship (‘thick’ citizenship) (criticised by Robottom and Hart [42]);
- The over-emphasis on education and awareness as the route to citizenship, with limited understanding of institutional barriers to citizenship participation; and
- A lack of understanding of the rights aspects of marine citizenship. There is a wide scope for further interrogation of who citizens are, what citizenship is, and what its role is in marine policy.

To progress a new understanding of ‘thicker’ marine citizenship this study explored how marine citizens themselves conceptualise marine citizenship and the range of responsibilities included therein. We also explored their awareness of the legislative background to environmental participation and marine citizen experiences of such participation, and consequences for perceptions of citizen empowerment. We considered the following research questions: how do marine citizens conceptualise marine citizenship; how do they perceive its role in marine governance; what are their experiences of public participation in environmental decision-making; and how aware are they of their legal rights to participate?

## 2. Materials and methods

### 2.1 Study participants

**Fig 1:**
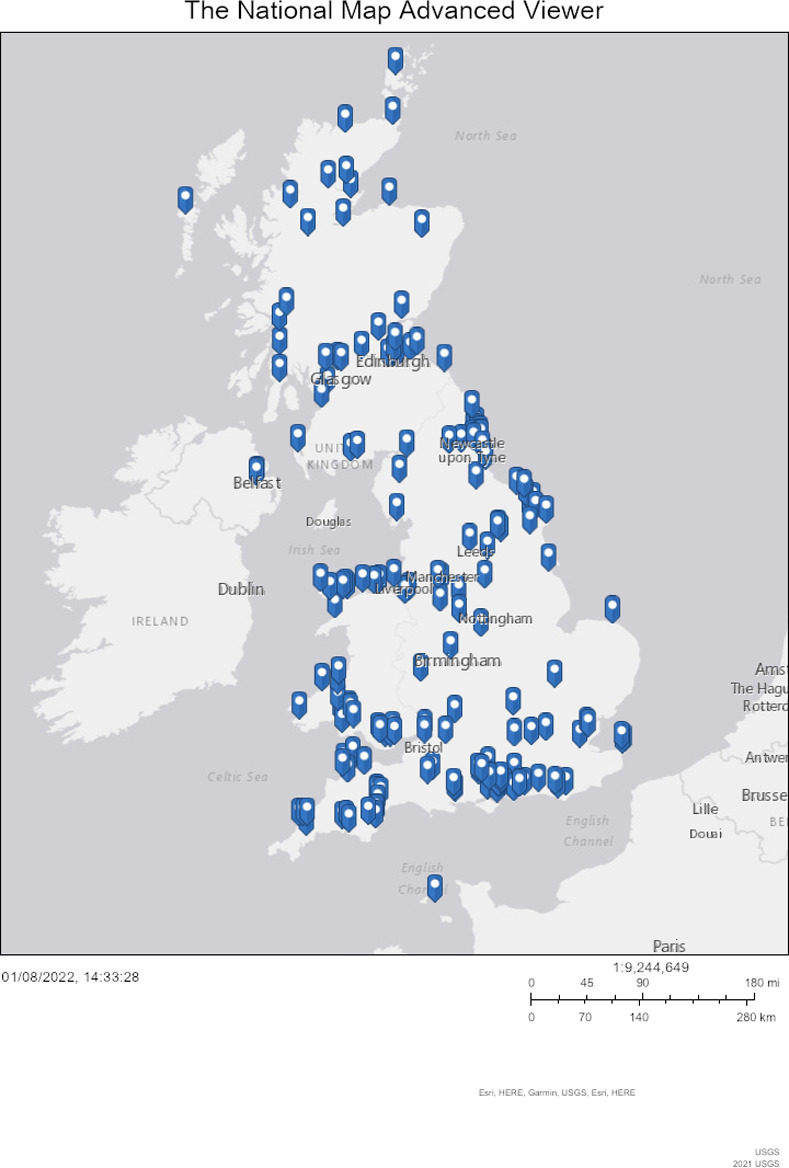
Geographical distribution of respondents. Distribution by partial postcode of online survey respondents from three case studies: a town-based marine group (n = 22), a regional marine group (n = 30), and a national marine citizen science project (n = 228). Markers indicate one or more respondents at each postcode location. Map constructed in USGS National Map Viewer.

The marine citizenship field has so far has been dominated by marine practitioner perspectives. In this study, we privilege the voice of marine citizens themselves. Study participants were all active marine citizens who were identified through three UK-based case studies. Two were community marine groups of differing scale and nature, in terms of demographics and typical activity, providing contrast in marine group composition, function, and ways marine groups can embed citizenship in policy-making. The third case was a national citizen science project aimed at engaging members of the public in documenting baseline rocky shore biodiversity data, called Capturing Our Coast (CoCoast, www.capturingourcoast.co.uk). The latter project enabled a nationwide response to the survey (Fig 1). Survey and interview participants provided written, informed consent.

### 2.2 Study design

Due to their advantage for collecting multiple viewpoints and perspectives [43], mixed methods were used to collect and analyse qualitative and quantitative data. Multiphase mixed methods were employed [44] in an iterative design incorporating, in sequence: two key informant open-ended interviews together with ethnographic shadowing of participants in their roles leading marine groups; an online survey collecting both quantitative and qualitative data (N = 280); eight further interviews with and shadowing of participants, purposely selected using survey data to create a diverse interview sample. This approach reflected a multi-sited ethnographic approach in which the concept of marine citizenship was followed as “a *slice of the world system*” [45, p113] and findings were triangulated across multiple datasets to improve their validity. The lead author and researcher conducting the fieldwork was more an ethnographer activist, embedded in the marine citizenship practice of the participants, than an objective observer.

The study was deductive and inductive, examining a wide range of factors known to be influential on pro-environmental behaviours and environmental intentions, but allowing space for new themes, factors and concepts to emerge from the data and lead further analyses. Factors purposefully investigated for this paper via the survey included multiple selection questions examining demographics, general and marine citizenship activity, and attitudes towards marine/environmental policy-making. Open questions in survey and interviews were used to reveal views about and experience of marine citizenship; knowledge of environmental participatory regulatory frameworks; and experience of marine decision-making.

This methodology provided a wide scope of findings, enabling a better understanding of how different variables interact and their relative importance, and how these relationships change across types of citizenship participation. The research was approved by the University of Exeter ethics board (2017/1452).

### 2.3 Data collection and analysis

#### 2.3.1 Quantitative

Quantitative data was solely collected by survey (see S1 File), which was delivered using Bristol Online Survey software, with a paper-based version for respondents without internet (n = 3), using single-stage, non-probability sampling [44]. The survey was delivered by email in October 2017 to all registered members of each case study which was approximately 120 for each marine group and 2800 for CoCoast. Responses were anonymous unless respondents indicated they wished to participate further.

**Fig 2:**
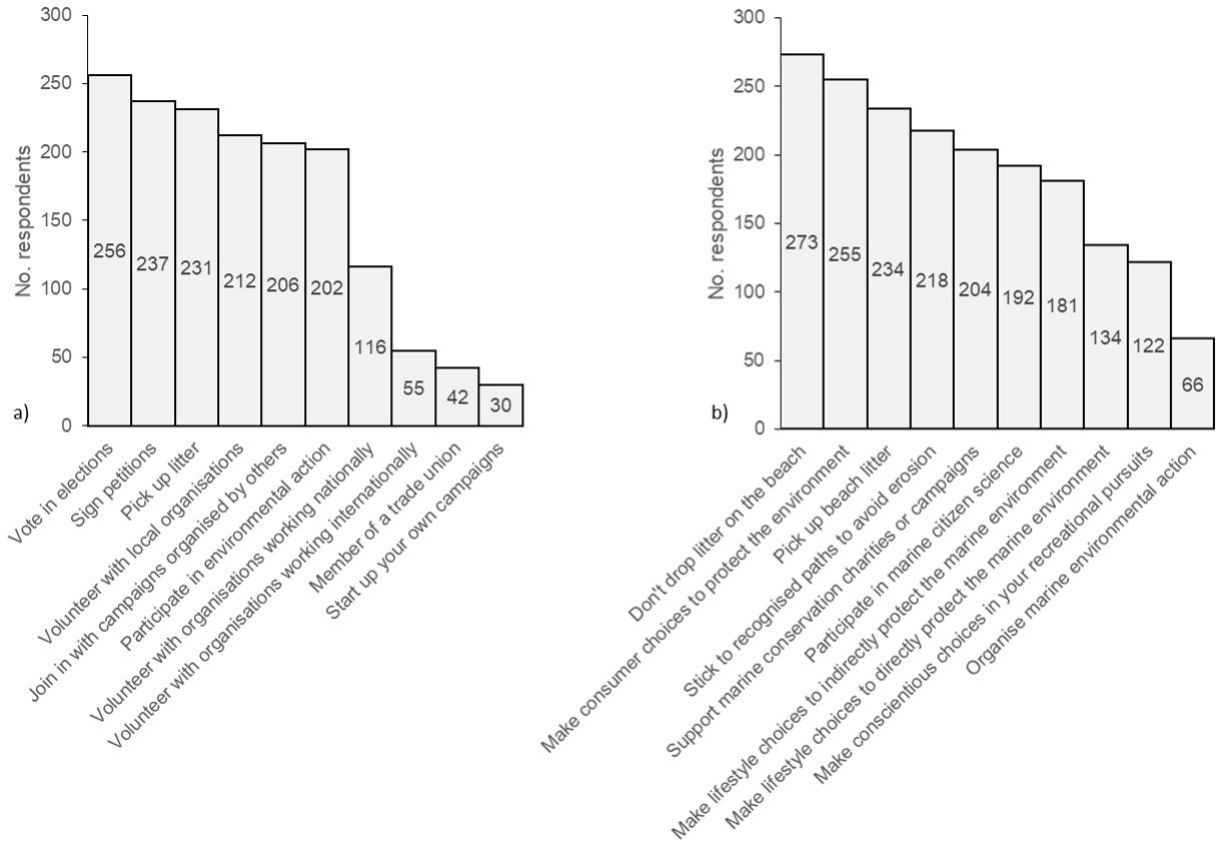
General and marine citizenship action frequency. Number of respondents performing a range of a) general citizenship and b) marine citizenship actions. Mean number of citizenship actions undertaken by respondents was 5.67, mean number of marine citizenship actions performed by individual marine citizens was 6.71, N = 280.

Basic socio-demographic data were collected via multiple-choice questions. General and marine citizenship participation was indicated via a sum score from a multiple-choice list of actions (see Fig 2 in Results).

A novel scoring metric was devised to measure depth or ‘thickness’ of marine citizenship. Items from the multiple-choice list were arranged into five categories reflecting increasing commitment (time, cost, or how public actions are) and focus on the marine environment specifically. The ordering was triangulated by the proportion of respondents doing each action on the assumption that easier and less costly actions would be done by more people (Table 1). Items within each category were averaged and the total possible score was therefore 15. The marine citizenship score was used in subsequent tests to examine influence of variables upon marine citizenship thickness.

**Table 1:** Marine citizenship scoring metric. Marine citizenship activities are categorised according to level of commitment and modification of life and given a score which increases with ‘thickness’ of the citizenship activity.

Score	Category	Marine citizenship activity	Percentage of sample participating
**1**	No action	Don’t drop litter	97.5
**2**	Active choice, fairly incidental	Consumer choices	91.1
		Pick up beach litter	83.6
		Taking paths to avoid erosion	77.9
**3**	Active commitment of time/money	Supporting marine conservation	72.9
		Participating in marine citizen science	68.6
		Lifestyle choices (indirect) e.g. energy saving	64.6
**4**	Active commitment of time/money with modification specifically for marine environmental health	Lifestyle choices (direct)	47.9
		Recreational choices	43.6
**5**	Proactively making change	Organising activities	23.6

Quantitative data were descriptively and statistically analysed in Microsoft Excel and IBM SPSS 25. ANOVA or Mann-Whitney U were used to investigate the strength of relationships and differences within and between the data sets [44].

#### 2.3.2 Qualitative

Open-ended survey questions were used to examine professional and educational experience; views on the utility of marine citizenship as a policy tool; actions considered to be marine citizenship additional to the multiple-choice question; experiences of marine-environmental decision-making; and awareness of rights concerning participation in environmental decision-making. Open-ended interviews were guided by a flexible protocol covering general marine citizenship practice; motivating and influencing factors; relationship with the sea; place, identities and values; and role and activities within the case study group. Two initial interviews were with key informants from the marine groups in July 2017. To add context to interview data [44], interviewees were shadowed as they participated in a marine citizenship activity of their choosing. These activities were beach cleans, marine group meetings, lobster hatchling release, commercial nature viewing boat trip, citizen science recording, and a public engagement event. The shadowing typically lasted 1–3 hours, and interviews held concurrently or immediately subsequently for 1–2 hours. Interviews were recorded and transcribed. Interviews and shadowing took place between July 2018 and December 2018.

Interview transcripts were initially coded by hand according to both themes relating to purposively investigated variables, and emergent themes, using common coding procedures [44, 46]. They were then reviewed, ordered and consolidated during transfer to NVivo 12, which was used for analysis.

Quantitative and qualitative survey data informed the interview design and sampling. To further enhance the mixed methods approach, quantitative variables were imported into NVivo to act as attributes for cross-analysis with interview coding. Similarly, quantitative analysis was performed on coding frequencies. Analysis was informed by emergent findings in both types of data. Neither data type was therefore privileged over the other.

## 3. Results

The survey elicited 280 responses with a response rate varying from ~8–25% with a higher rate in the marine groups. Survey respondents were 60.4% female, 37.9% male, and 0.4% transgender. Age ranged from 19–82 years. Ten survey respondents were further involved in the interview and shadowing phase.

The results are presented to respond in turn to each research question:

How do marine citizens conceptualise marine citizenship: In 3.1 we present marine citizens’ conceptualisations of marine citizenship, interrogating marine citizenship responsibilities both as private pro-environmental behaviours (‘thin’ citizenship) and as more public-facing actions (‘thick’ citizenship).

How do they perceive its role in marine governance: In 3.2 marine citizens’ views of the importance of marine citizenship are presented, and we argue that marine citizens intuitively include participation in marine policy-setting and decision-making, through both formal and informal participatory processes, as integral to their marine citizenship. Responding to this finding, we directly interrogate marine citizenship as participation.

What are their experiences of public participation in environmental decision-making: In 3.3 we present marine citizens’ experiences of participation in marine decision-making, both formally and informally, and their perceptions of how efficacious individuals and other groups are in marine decision-making processes.

How much do they know about these legal rights: We then contextualise marine citizens’ perceptions and awareness of the regulatory setting of procedural participatory rights (3.4) and environmental legal redress (3.5).

### 3.1 How do marine citizens conceptualise marine citizenship?

Multiple choice and open questions provided data on how our sample of marine citizens understood their own marine citizenship practices. Respondents were asked to select which general and marine citizenship actions they participated in (Fig 2). These selected actions were intended to provide a concise, quantitative measure of general and marine citizenship, including easily recognisable ‘thin’ and ‘thick’ actions, giving a range from pro-environmental behaviours (e.g., *pick up litter*) through to actions designed to change society (e.g., *organise marine environmental action*). Marine citizens were found to be generally active citizens, for example, compared with a 68.7% general election turnout in 2017 [47], 91% voted and 85% signed petitions. The sum of **general** citizenship actions was positively associated with depth of **marine** citizenship: F(1,277) = 18.962, p < .001, B = 0.161, adj. R^2^ = 0.061. The 22.9% Green Party political support in the survey population contrasts with the 2017 UK general election 1.6% Green Party vote share, indicating an association between environmental issues and politics in marine citizens. Taken together, these findings provide evidence for marine citizenship as part of wider political understandings of citizenship. However, on average, marine citizens did more marine than general citizenship activities of those listed, indicating that the marine setting of this kind of citizenship is important.

The most common marine citizenship actions were those most accessible such as beach cleaning and citizen science: “*Beach cleaning you get every age*. *You’ll get little tiny kids*, *teenagers*, *retirees*, *people who are working*”. Unsurprisingly in a population largely derived from a citizen science project, this too scored commonly amongst respondents, however many other projects and databases were referenced by respondents: wildlife recording; nurdle and litter recording; and a range of formal recording schemes. Citizen science was highly regarded as creating scientific and legitimate knowledge to improve research and policy-making, and to create an historical record.

In an open text box, respondents identified marine citizenship actions they considered to be different from those in the multiple-choice list. Many of these activities mirrored the multiple-choice actions (Fig 2), but others indicated an important social capital dimension to marine citizenship (Table 2).

**Table 2:** Additional marine citizenship actions. Additional actions identified by marine citizens in a free text question indicate an important social capital dimension to marine citizenship.

Theme	Number of references	Number of respondents	Detail
**Champion**	91	89	Public engagement as a champion of the ocean.
Ambassadors	(78)		“*Spreading the word*”
Engaging children	(11)		Others’ and own children
Artistic output	(2)		
**Professional output**	19	14	Professional role, collaborative working, teaching, artistic output, and professional participation in decision-making. Distinct from volunteering.
**Stewardship**	10	10	An active role as a steward of the marine environment by reporting incidents or challenging others on their actions
**Learning**	6	6	Acquisition of knowledge as an act of citizenship–informed citizen.
**Marine conscience**	4	4	Presence of a conscience about the marine environment rather than specific actions

Marine citizens expressed a strong sense of citizenship responsibility and moral duty, evidenced by a range of voluntary and community activity. For some the marine aspect was an interest-based means of satisfying that responsibility, often in amongst other general citizenship actions, for example: *“The citizenship bit*, *the volunteering*, *the contributing*, *the noblesse oblige*, *is really not a lot to do with the marine world”*. Responsibilities were to clean, to assuage guilt, to share nature with others, to learn, and to inform others. E.g., “*I think citizenship is about taking part*, *keeping your own knowledge up to date*, *sharing that with other people*, *helping reach out to different groups of people”*. *Lobbying* arose as a theme in the interviews, representing political and civic environmental action and intersected with themes such as *stewardship* and *marine conscience*. The data collectively indicate an important intersection between marine citizenship and wider civic participation.

Marine citizens’ perspectives on marine citizenship challenged existing understanding of the concept as an information-driven set of pro-environmental behaviours (i.e., if the public understand the problem they will change their behaviour to be part of the solution and, thus, become environmental citizens). In this study, marine citizens recognised a wider range of roles for knowledge including as a barrier or enabler of marine citizenship; learning as an act of marine citizenship; public engagement and environmental literacy of others; and as important for policy development and decision-making (Section 4.2). Additionally, it was noted that marine groups were viewed as an important vehicle for knowledge exchange and facilitating these applications of knowledge.

Whilst respondents did talk about knowledge building or sharing as being a motivator of **others’** marine citizenship, e.g., “*People having an understanding of the marine environment ensure[s] they are likely to help it”*, respondents did not cite information or knowledge as being the *motivator* for their **own** marine citizenship, rather they described it as an *enabler* supporting their existing motivation to become active. Only 3.6% of respondents cited their lack of knowledge as a barrier to marine citizenship. This suggests the normative understanding of a linear relationship between education and environmental behaviour change is widely adopted and perpetuated, including by active marine citizens themselves, despite marine citizens drawing on a broader set of factors, such as emotional connection to the sea, as their own motivators for marine citizenship [48].

Local environmental and place knowledge was considered important. Respondents talked of a connection with place incorporating a knowledge and understanding of it, which in turn would drive the motivation to take action to protect that place. There was a sense that local place attachment would lead to interest, learning, and then marine citizenship: “*Teach them about passion in the area that they live in first and then explore further afield*”. Indeed around a third of survey respondents (n = 89) cited learning as a marine citizenship action and as a motivation for becoming involved in their marine group or project.

Public engagement by raising awareness, sharing enthusiasm, and facilitating learning in others was a prominent marine citizenship action (Table 3). Audiences for these exchanges ranged from family and friends to strangers, via formal events or incidental conversations. Marine groups and public marine citizenship activities, such as recording wildlife in public spaces, facilitated these exchanges, which were intended to help people learn and to engage them in the qualities of the ocean in different ways.

**Table 3:** Public engagement as marine citizenship. Examples of qualitative data relating to public engagement as an action of marine citizenship, as cited by a population of active marine citizens.

Public engagement purpose	Example data
**Aesthetic qualities of the sea**	*“Sharing the beauty of the coast on social media in regular photos”*
	*“I record photographically the sea scape so that others will not forget what we are fighting for”*
**Wildlife protection**	*“Have put up posters alerting public/dogwalkers to ringed plover nesting site on our local beach”*
**Wildlife as intellectually interesting/beautiful**	*“Help to educate others about the seashore life”*
	*“Just posting photos on Facebook of the amazing sea creatures I find*, *to show my friends what’s out there”*
**Marine citizenship actions**	*“Encourage others to make more eco-friendly life choices about food*, *types of cleaning products*, *recycling*, *picking up litter etc*.*”*
	*“Pass on information that isn’t widely known to friends and family to influence their consumer and lifestyle choices”*
**Human threats to the sea**	*“Teaching children about the marine environment and about the dangers of marine litter*.*”*
**Environmental values**	*“Inspire others to value the sea and become involved in marine conservation activities”*
**Environmental education**	*“Educating my kayaking customers about the place and environment as well as the activity they’re doing”*

### 3.2 How do marine citizens view the role of marine citizenship in marine governance?

We asked survey respondents “*In what ways do you think marine citizenship is important for marine environmental health*?” in order to understand how marine citizens saw the value of their citizenship for marine governance outcomes. Responses referred to the social capital impact of marine citizenship more than specific environmental outcomes (Table 4). Political understandings of citizenship emerged through a view of collective action as an effective agent of change, though it was implicit that the action was often derived from awareness raising and public education.

**Table 4:** Role of marine citizenship in marine environmental health. Responses given by active marine citizens to the question “In what ways do you think marine citizenship is important for marine environmental health?” Responses were coded to draw out key themes. (N = 249). NB. Within *Responsibility* is included sub codes of Universalism value, Caring and Ownership which were all connected with an expression of being universally responsible for marine environmental health. Example data is provided for most-referenced themes. *Important* was coded where responses using language stressing that marine citizenship is important/vital and similar.

Code	Example data	No. coding references
**Awareness raising**	*“Helps spread awareness of what needs to be done*.*”*	[71]
	*“Greater awareness of factors affecting the marine environment might make changes to your habits*.*”*	
**Applied knowledge**	*“By understanding our environment better we are more able to protect and preserve what we have and even help with regeneration of particular systems*.*”*	[52]
	*“The more data we can collect the better and more informed the decisions*.*”*	
**Knowledge**	*“The marine environment is under threat through ignorance of its importance and marine citizenship is a way to understand and get closer to the environment*.*”*	[45]
	*“The more people that know and care about the marine environment*, *the more likely it is to be protected*.*”*	
**Responsibility**	*“Community engagement and knowledge makes everyone stakeholders and gives common responsibility*.*”*	[43]
	*“Promoting personal responsibility and changing attitudes is crucial to creating the idea that we are custodians of the environment*.*”*	
**Collective action**	*“If more people were to be involved*, *hopefully we could reduce and improve environmental impacts*.*”*	[29]
	*“It is important to be a member of a group or community to make your voice heard*.*”*	
**Important**	*“Extremely important if members of the public don’t get involved in marine citizenship activities then we can’t conserve our marine environment*.*”*	[23]
**Marine health**	*“The more litter picked up*, *the less there will be on the beaches and in the sea*, *the less birds and animals will ingest and get caught in*.*”*	[22]
	*“Ocean health depends on people acting appropriately and agitating for politicians to make appropriate policies—this demands an informed populace—marine citizens*.*”*	
**Behaviour change, Participation, Campaign, Empowering, Encourage others, Necessity, Small consequence, Apathy**		<20

When asked specifically about the value of marine citizenship to marine decision-making, *citizen empowerment*, *informed decision making*, and relative *power balance* of different actors were the most prominent themes (Table 5). This again reflected the political interpretation of marine citizenship amongst marine citizens, seeking to create change through social action and knowledge exchange.

**Table 5:** Importance of marine citizenship in marine decision-making processes. Responses given by active marine citizens to the question “In what ways do you think marine citizenship is important for the process of marine decision-making?” Example data from sample of active marine citizens (N = 207).

Code	Example data	No. coding references
**Citizen empowerment**	*“Citizen actions influence decisions*. *Marine citizenship can even instigate decisions”*	[69]
	*“It gives a voice to those directly impacted by decision”*	
**Informed decision-making**	*“Having enthusiastic and dedicated people who spend a lot of time in the area provides up to date data on the state of the marine environment*.*”*	[51]
	*“Informed decision making is key to good policy*. *It isn’t always the obvious thing that’s best*.*”*	
**Power balance**	*“I think marine environmental groups do not have enough say in marine decision making*.*”*	[36]
	*“We are more down to earth*, *less captured by the self-interest*, *ethos and jargon of the professionals”*	
	*“The sea and shore belongs to use all… We should all had a say in even the smallest decisions*.*”*	
**Raising awareness**	*“It’s the main form of communication to most general citizen about marine policy decisions*.*”*	[18]
	*“Raising awareness*, *encouraging active participation*.*”*	
**Knowledge deficit**	*“People are better able to express opinions and have their say if they have a solid understanding of what it is they’re trying to protect*.*”*	[13]
	*“The more the public is educated the more influence they can have”*	
**Local knowledge**	*“In coastal waters*, *many locals understand the oceans better than those writing the policy*, *and their views on how proposed changes can effect both the environment and local businesses are vital*.*”*	[13]
	*“Local people know their area and are directly affected by decisions and so should be a big part of the decision making process”*	

A Mann Whitney-U test was used to examine the difference between those who had and had not participated in marine decision-making according to their general and marine citizenship scores. The data revealed that those with deeper citizenship scores, in particular deeper marine citizenship scores, were more likely to have participated directly in formal marine decision-making processes (Table 6). This association adds quantitative weight to our proposed inclusion of civic participation within the marine citizenship concept.

**Table 6:** Relationship between participation in marine environmental decision-making and citizenship scores. Participation in marine environmental decision-making (yes vs no) according to general citizenship score and marine citizenship score, via Mann Whitney-U.

Variable	U	z	Asymp. sig.	Median		Factors associated with participation in marine environmental decision-making
Citizenship score	11,827.0	6.520	< .001	Yes	7.00	More general citizenship activity
				No	5.00	
Marine citizenship score	11,360.5	5.680	< .001	Yes	10.00	More marine citizenship activity
				No	6.17	

In sum, marine citizens are not limited to private actions of marine citizenship, but engage in the public sphere to promote education and changed attitudes in the wider public. Working collectively to develop an informed and interested public, and promoting civic participation in environmental matters, situates marine citizenship much more politically within the environmental social movement and challenges normative individualistic understandings of it.

These results signpost to a marine citizenship concept which is wider than individual actions, and which uses social strategies to effect change within political and decision-making institutions. Through this interpretation, it is logical to understand the rights aspect of marine citizenship as being less about substantive environmental rights, than about political and civic rights to participate in the transformation of the human-ocean relationship, including its management by the state.

### 3.3 Marine citizens’ experiences of exercising their right to participate in decision-making

Eighty respondents (28.6%) had engaged in marine decision-making through a variety of means, of which 78 provided information about their engagement (Table 7). This was predominantly via formal consultation, of which half related to the designation of the UK’s Marine Conservation Zones (a Marine Protected Area (MPA) designation under s.116 of the Marine and Coastal Access Act 2009 [49]), indicating that there has otherwise been limited opportunity, or awareness of opportunity, to directly shape the use and governance of the marine environment in the UK.

**Table 7:** Participation in marine decision-making. Summary of types of participation in marine decision-making from a group of active marine citizens. N = 78. Some respondents detailed more than one activity. Respondent perception of efficacy of the participation is provided for each activity type, where that information was provided, together with a percentage proportion of those data. For ease, bolded figures indicate the majority outcome for each activity type.

Participation activity	No. coded references	Efficacy of participation		
		Positive	None	Unknown
**Consultation**	52	**17** (42.5%)	13 (32.5%)	10 (25.0%)
Marine Conservation Zone consultations	(27)	**8** (38.1%)	6 (28.6%)	7 (33.3%)
Other general environmental consultations	(20)	6 (37.5%)	**7** (43.8%)	3 (18.8%)
Other marine environmental designation consultations	(5)	**3** (100.0%)		
**Citizen Science**	13	3 (33.3%)	3 (33.3%)	3 (33.3%)
**Petitions**	12	3 (42.9%)	3 (42.9%)	1 (14.3%)
**Planning**	12	4 (44.4%)	4 (44.4%)	1 (11.1%)
**Professional engagement**	11	**5** (62.5%)	2 (25.0%)	1 (12.5%)
**Lobbying elected representatives**	11	**4** (40.0%)	3 (30.0%)	3 (30.0%)
National Government	(8)	2 (28.6%)	**3** (42.9%)	2 (28.6%)
Local Government	(2)	**2** (100.0%)		
European Government	(1)			**1** (100.0%)
**Campaign (inc. NGO-led)**	7	2 (40.0%)	**3** (60.0%)	
**Public engagement**	3	1 (50.0%)	1 (50.0%)	
**Coastal partnership**	2	1 (50.0%)		1 (50.0%)
**IFCA Byelaw creation**	1		**1** (100%)	
**Marine Education policy development**	1	**1** (100%)		
**Total**	125	**41** (43.6%)	33 (35.1%)	20 (21.3%)

67 respondents included information about the efficacy of their participation allowing it to be coded as having a positive impact, no impact, or unknown impact on a particular marine decision-making process. When disaggregated, the sample sizes for specific activities become small, however there is an overall picture of more positive experiences for those directly engaged and those engaging on more local scale actions, e.g. through consultation or lobbying local elected representatives. There was a more positive view where the process was perceived as fair and outcomes were communicated, even if they were not favourable to that person’s view because “*They were listened to*”. Despite their limitations in participatory quality (50), consultations usually have a visible outcome, for example the MPA gets designated.

**Fig 3:**
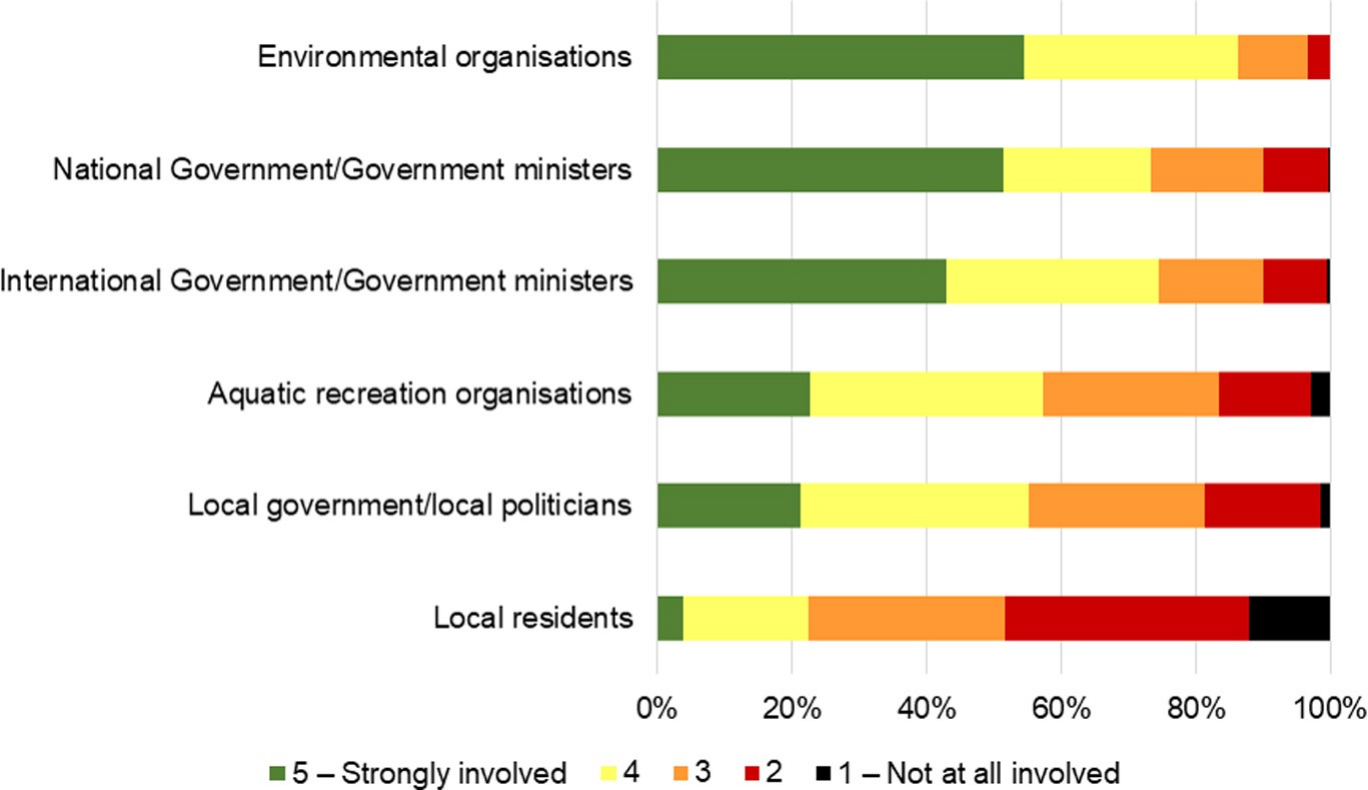
Empowerment in marine decision-making. Responses given by active marine citizens to the question "To what extent do you think each of the following are involved in marine and coastal decision-making?”. Chart shows Likert score for each category on a 5-point scale from not at all involved (1) to strongly involved (5). N = 280.

Though local participatory opportunities appeared more frequent or accessible to marine citizens than opportunities at larger scales, the power to create environmental change was perceived by marine citizens to be concentrated at larger scales (Fig 3). National and international governments and NGOs were perceived to have more influence than individuals and local governments. This is tacit indication that the public representation role of NGOs legislated for in the Aarhus Convention and related legislature is recognised by marine citizens, even if implicitly. Interviewees expressed support for NGOs: “*I think that the NGOs…have an effect… they’re one of the best ways for the people who care*”.

Perceptions of grassroots empowerment may be negatively impacted by procedural rights to participate in environmental decision-making being granted above the level of the individual, through NGOs for example. The lack of individual empowerment was discussed by the interviewees, particularly those who were under 45 years old and those engaged more directly with policy-making. Marine citizens advocated for a grassroots voice: “*It’s hugely important that people at grassroots level have a voice*, *and the voice is then listened to*, *and it’s added to*, *and the momentum continues*, *because then the politicians have to listen*.” And recognised the democratic legitimacy of a public voice: “*If you could change the policy then that is the quickest way to make a change*. *But you’ve kind of got to have people on-side to do that*…*The policy’s not going to change unless people want it to change*.” There was also real excitement about the development of marine and more general environmental citizenship: *“[online petition sites] had a lot of lobbying success with the huge multi-nationals and things*, *so I think it’s the most exciting time…since the printing press*! *For…global citizenship*, *finding other like-minded people and forcing governments to effect change*”.

### 3.4 Marine citizens’ understanding of public participation legislation

We wanted to understand what awareness marine citizens had of existing rights to such participation. We asked survey respondents, through an open text box: “*Are you aware of any international/EU/national legislation which promotes the processes of citizenship and public participation in environmental and marine decision-making*? *Please describe what you are aware of*?*”* The question elicited 28 individual legislative instruments and policies from domestic to international scales (n = 38, noting 26 had marine or environment/general science degree and 15 had or did work professionally in the sector) (Table 8).

**Table 8:** Public participatory procedural rights. Awareness of environmental public participation legislation and policy in a population of active marine citizens. n = 38.

Legislation/Policy	No. of references	Nature of public participation
**Marine and Coastal Access Act/Marine Conservation Zones (MCZs)**	21	Marine planning
		MCZ consultation
**Aarhus Convention (related EU Directives)**	4 (2)	Public access to environmental information, justice, and participation in decision-making
**UNESCO**	3	Involvement of local communities
**UK planning law**	3	Consultation requirement
**Environmental Impact Assessment**	1	Consultation
**Strategic Environmental Assessment**	2	Consultation
**SUSCOD**	1	Integrated coastal zone management
**RIO Declaration**	1	Participation of citizens concerned
**18 environmental policies**	38	Related to environmental protection or conservation

Strikingly, 131 of the 280 survey respondents stated they did not know of any instruments and 74 left the question blank. Awareness of the right to participate in environmental matters was very low. Awareness was weighted towards environmental protection legislation rather than public participation provisions. The evidence indicated that the Marine Conservation Zone consultations, cited by 21 respondents, have had a wider public reach compared to other instruments. There was awareness of the challenges of reach via consultation: “*all* [policies] *state requirements for consultation with stakeholders*, *wide dissemination*, *but in reality most are unclear about who they define as ’public’ and ’stakeholder’ and at what stage and how they should feed in*”. This represents a paradox where formalised consultative participation has greater reach, yet offers only a weak form of participation for citizens [50].

### 3.5 Environmental legal redress

The final area of investigation of marine citizenship rights was awareness of access to procedural environmental justice. Survey respondents were asked “*If you are not happy with an environmental decision from a regulatory body and/or law or regulation*, *which avenues are available to you for legal redress*? *Please also describe if you have ever used one*, *or how else you can raise your concerns*. *If you don’t know*, *please state so*.” Of 280 respondents, 94 said they didn’t know and offered no suggestions and 69 left the question blank. The suggestions of the remaining 115 respondents who made statements are presented in Table 9. Elected representatives were the most common response, followed by organised campaigns such as petitions. Additionally, some respondents cited NGOs as taking on legal battles and recognised them as representative of the public: “*I would say they would represent people…How else would they be represented*?” NGOs were however identified by respondents as facing challenges such as funding instability and inter-NGO competition which may compromise their effectiveness in representing the public effectively or fairly.

**Table 9:** Awareness of environmental justice. Means of seeking environmental redress proposed by a sample of active marine citizens. (n = 115).

Code	Example data	No. of references/respondents
**Elected officials/government bodies**	*“I expect one can write to ones MP or Minister in charge of a department but would not hold out much chance of success”*	[64]
(Local, devolved administration, national, EU)	*“Contact and write comments to local council if plans open for viewing*. *Contact local MP”*	
**Campaign**	*“I can create a petition for my local councils or to be reviewed in parliament*.*”*	[38]
(Petitions, protest, write letters, lobby, social media, traditional media)	*“Media cover*, *signing petitions*, *organising Public meetings*.*”*	
**Regulatory body**	*“The options available depend on the regulatory body / law involved*.*”*	[15]
	*“Regulators normally have a public complaints system*.*”*	
**Legal advice/action**	*“During our period of fighting the raw sewage proposal I was aware that legal support would be available if the decision went against us*.*”*	[13]
	*“Judicial review*. *Currently in the process*.*”*	
**Participation in decision making**	*“Have used all the steps in planning processes up to and including speaking at a public enquiry—at local authority level and at national infrastructure (Development Consent Order) level”*	[6]
**NGO:**		
Local	*“I haven’t had to*, *but if I did my first point of contact would be the most active NGOs in the region*, *especially if I knew they had already been involved in the consultation process”*	[15]
National	*“Probably look to act through organization*, *e*.*g*. *green peace”*	[16]
International	*“Local*, *national; and international environmental organisations”*	[1]

16 respondents volunteered factors acting as barriers to legal redress, such as cost of legal representation and limits to who is considered affected by a matter and able to access procedural redress. One respondent felt lack of knowledge prevented their access to justice: “*As a diver*, *I see a number of things that concern me and other members of the diving club I belong to*. *But I don’t know who to voice these concerns with*.” There was also cynicism about how much public views can influence regulatory action and decision-making. Overall there is a lack of awareness about access to environmental justice amongst marine citizens, who actively seek to inform themselves on marine issues and who are particularly active civic participation. It would not be unreasonable to imagine that the wider general public would be less well informed of these rights.

## 4. Discussion

This paper presents an analysis of marine citizens’ conceptualisations of marine citizenship and characterisation of marine citizenship rights. The findings give insight into how marine citizens conceptualise marine citizenship, including its importance for marine policy and decision-making; and a novel and important exploration of marine citizenship as a right to participate in the transformation of the human-ocean relationship, including ocean management. Here we discuss the insight these findings give into how marine citizenship intersects with marine policy and management, and we propose an extended definition of marine citizenship.

### 4.1 Marine citizenship

Mirroring the language of environmental law and citizenship theory [26, 51, 52], the understanding of marine citizenship by marine citizens was much ‘thicker’ than the individual pro-environmental behaviours most commonly discussed in the literature [17, 18, 29, 30, 53], relating more to a life lived than a set of choices limited to individual environmental impact.

Marine citizens acknowledge the environmental impact of their individual actions, and some would agree this is a primary cause of environmental impact [54]. Yet, they also acknowledge the institutional and social context of environmental harm and articulate a commitment to collective action via civic participation, learning and public engagement to address this. These views support our proposition that marine citizenship is more than ‘thin’ private and individualised actions as it incorporates deeper public-facing and collective action. As a socio-political act, the concept is opened up to the wealth of knowledge and understanding that scholars of citizenship can offer.

The focus on awareness raising and knowledge as a reason why marine citizenship is important for marine environmental health, provided evidence that as a group, marine citizens had internalised the prevailing knowledge deficit approach to behaviour change, aligning with previous studies [14, 17, 26, 28, 29, 54–56, *inter alia*]. However, though marine citizens frequently referred to knowledge as a motivator of marine or environmental citizenship in *others*, particularly through public engagement activities, knowledge was expressed as an enabler of *their own* marine citizenship, and not a motivator. Here, marine citizens implicitly demonstrated the importance of the first pillar of the Aarhus Convention [35], which relates to access to environmental information. Research has postulated lifelong learning as an act of political citizenship [57, 58] and this position was certainly represented amongst this population of marine citizens. Educating oneself and the wider public increases social capital and supports an informed democracy, and it is in this wider context that knowledge exchange was primarily understood by marine citizens.

Public-facing actions make marine citizens more vulnerable than private actions, yet the emergence of public engagement as a key marine citizenship activity indicates a goal to increase community and political participation of the civil society [59]. This has two key implications, first that future iterations of the marine citizenship score need to include public engagement actions, and second that marine policy should be concerned with a much thicker framing of marine citizenship to maximise its benefit to the marine environment. This can be supported through policy decisions such as broadening ocean literacy to include political and civic literacy. Such concrete knowledge of participation positively predicts environmentally responsible behaviour [56].

### 4.2 The role of marine citizenship

Having established that marine citizenship is much thicker than private, individual actions, it becomes easier to see the potential utility of marine citizenship in engaging civil society, promoting civic participation, and knowledge exchange. Actions such as citizen science and public engagement were viewed as particularly valuable for incorporating local knowledge into decision-making and contributing to the scientific evidence base via citizen science. Better integrating local and scientific knowledge in decision-making can help address issues around public valuation of scientific opinion [60] and legitimacy and acceptability issues arising from over-privileging scientific knowledge in marine planning processes (e.g., Marine Conservation Zones: [52]). Legally, knowledge quality of participants must not be predetermined, giving un-due influence by those in authority over the procedure [41]. Citizen science debates are already addressing issues such as quality assurance of citizen-acquired data [30], and scientific rigour of citizen science [e.g., 61–63]. Adoption of pluralistic and interdisciplinary approaches to knowledge by practitioners and policy-makers produces better marine governance outcomes [64, 65].

Of particular value, was the marine group structure, acting as a source of opportunity and social experience. Marine groups were viewed as a support for marine citizens through a like-minded community, and an effective means of engaging with existing policy structures. Marine groups served to raise social capital and collective empowerment and thus widen the possibilities of marine citizenship having positive impact upon the ocean directly and through changing policy. All people can potentially participate in thick marine citizenship regardless of their proximity to the coast through collective and political action.

### 4.3 Marine citizenship rights

As well as re-characterising marine citizenship, a key contribution of this study is the investigation of marine citizenship as a right, addressing a key gap in the literature. The strength of expression about public engagement and empowerment, together with their valuing the contributions publics can make to policy and decision-making, are a clear indication that, for marine citizens, marine citizenship means participation in “*the process of construction and transformation*” [p53, 66], going beyond the narrower and normative view centred on behaviour change we have interrogated here.

Despite being some of the most engaged people, some professionally, marine citizen awareness of public participation and environmental justice legislation was very low, as has been found in other nations [67]. Even these active marine citizens felt that individuals are disempowered in exercising these rights, with access to justice limited by knowledge and cost, supporting arguments made that there is a lack of empowerment in environmental justice [39] and that costs are one of the barriers [68]. It is difficult to say how this lack of awareness of environmental procedural rights may impact upon the potential for marine citizenship to contribute to a healthier marine environment. We would recommend that this would be an important area for future research. From a policy perspective, marine citizenship might be more effectively supported if formal environmental education includes this information, and ocean literacy practitioners consider what role they can play in improving environmental political literacy.

Despite the lack of awareness of the legislation itself, the impact of the Aarhus Convention in giving rights to the public particularly via environmental NGOs was felt through such organisations being viewed as empowered to create change and seek justice in this and previous research [18, 54]. Though in international law there are Conventions that contemplate participation in decision-making for transboundary projects, such as the Espoo Convention [69], these are narrower in focus and approach compared to Aarhus, and there remains a question mark over how the Convention will relate to larger scale impacts such as climate change or ocean degradation [40] which are administratively transnational and concern global commons. Through this research it’s clear that marine citizens would welcome access to be more politically involved in these challenges.

In the UK policy landscape, the Marine Conservation Zone consultation appears to have been a particularly prominent piece of public participation in marine planning. Here we found that local processes are more commonly accessed and more favourably viewed when compared to wider scale processes particularly due to participants receiving feedback on the outcome of the process. The MCZ consultation enabled more subsidiarity of a national process than has been typical in UK government environmental planning. There is still room for improvement in the design, given criticisms of the process charged at limiting the publics accessing decision-making, and purposeful stakeholder groupings exacerbating conflicting interests [52]. Alongside development in specific marine environmental proceduralisation, it should be recalled that barriers to marine citizenship, common to other forms of civic participation, will reflect wider systemic architecture which could be improved [15, 70, 71], particularly the potential role of marine groups to support marine citizens.

This analysis of marine citizenship rights adds a crucial piece to the puzzle of how marine citizenship can be an effective policy tool for tackling ocean degradation. Through interdisciplinarity, environmental law research can be drawn upon to inform how marine policy can be effectively harnessed to facilitate access to marine citizenship and improve effectiveness of individual and collective political action for the ocean. Marine practitioners can reflect on these findings and how they might be integrated into ocean literacy interventions.

### 4.4 Marine citizenship–a new definition

In this paper we have made the case for revisiting the normative approaches to marine citizenship as a set of individualistic and private pro-environmental behaviours that can be encouraged through public education and awareness raising. Without undermining the importance of these actions as a tool to better equip environmental citizens for civic participation [16, 31, 72, 73], findings represented here argue for a much wider definition of marine citizenship which acknowledges its political aspect, both for individuals and as a collective and public movement. Marine citizens spoke eagerly and passionately of empowering communities and grassroots, engaging the public and increasing social capital, and of local coastal place-making. This framing resonates much more with the collective action of social movements [74, 75] than with individualistic processes.

Our findings identify a huge untapped potential for a powerful marine citizenry to effect change on political structures that have to date been resistant to the transformative change necessary for sustainability. To fully realise the potential of marine citizenship there is a need for considerable growth in research across disciplines, in particular procedural and informal access to the right to participate.

To support the flourishing of further research, we have refined the current definition of marine citizenship to give more weight to rights and to the political context of citizenship, as follows:

*Marine citizenship is exercising the right to participate in the transformation of society’s relationship with the ocean*, *and acceptance of responsibility to make informed decisions and choices about personal and collective actions that will contribute to a sustainable marine environment now and into the future*.

This may be usefully operationalised as: *exercising the right to participate in the transformation of the human-ocean relationship for sustainability*.

This new definition challenges academia to answer questions about how this right to participate can be expressed to the public, what is necessary to effectively deliver on this right, and how marine citizenship can effectively lead to transformation. It also asks marine citizens to be informed, responsible, and exercise their rights. This new definition moves away from reducing individual impacts to committing to reshaping the human-ocean relationship. We recommend that future marine and wider environmental citizenship research builds on our first characterisation of marine citizenship rights to increase our understanding of how these can be supported by policy, and how accessibility and motivation to participate in decision-making might be developed.

### 4.5 Limitations

The study presented in this paper is limited in geographical scope to the UK policy and cultural landscape and is an exploration of members of the public who are already active marine citizens. Whilst participants were recruited from a limited number of case studies, these were a gateway to access active marine citizens and responses were not limited to experiences formed in the context of the case studies. As a novel metric, the marine citizenship score protocol will benefit from replication and refinement, and development for use in evaluating interventions in practice.

We note that online surveys can be problematic for sampling for example drawing respondents from online communities [76], however in this study participants were all grounded in ‘real-life’ local organisations and projects. Though self-selection can bias a representative sample, this study investigated a particular population of people who had self-selected to be active marine citizens.

## 5. Conclusions

In this paper we have presented the findings of an interdisciplinary, mixed methods investigation in the concept and practice of marine citizenship. We have expanded the knowledges used in marine citizenship research and have drawn on conceptualisations from marine citizens themselves. Through this approach, we extend previous understandings of marine citizenship as a set of private and individual pro-environmental behaviours, to include public and political acts of collectivism and public engagement. We argue that ocean literacy can support marine citizenship by engaging with political and civic literacy and supporting would-be marine citizens in accessing their marine participatory rights.

The role of knowledge has been contextualised to highlight how lifelong learning can be a citizenship act, and how knowledge acts as an enabler of marine citizenship, rather than a direct motivator. Both local and scientific knowledge is particularly valued by marine citizens as important for developing good marine decision-making.

This paper has presented a broad, novel analysis of the civic and political rights of marine citizenship, complementing the wider literature concerned with the social right to healthy environment. Such rights have been examined against the backdrop of existing environmental, participatory and justice focused, procedural legislation.

Through this new framing of marine citizenship using the language of citizenship, an updated definition of marine citizenship is proposed with which to challenge the scientific community and the public to go further and look wider at the potential of marine citizenship to create transformative change. We believe these findings will be of interest to a wide range of scholarly disciplines and practitioners who are concerned with environmental civic participation, environmental and ocean management, environmental education, pro-environmental behaviours, geographies of the sea, environmental psychology, and social-ecological systems.

## Supporting information

S1 File Survey questions. Online and print survey used in this study to investigate marine citizenship and a range of factors influencing it, in a population of marine citizens.
(PDF)

## References

[1] Costanza R, de Groot R, Sutton P, van der Ploeg S, Anderson SJ, Kubiszewski I, et al. Changes in the global value of ecosystem services. Glob Environ Change. 2014 May 1;26:152–8.

[2] Fleming LE, Maycock B, White MP, Depledge MH. Fostering human health through ocean sustainability in the 21st century. People Nat. 2019;1(3):276–83.

[3] Pauly D, Watson R, Alder J. Global trends in world fisheries: impacts on marine ecosystems and food security. Philos Trans R Soc B Biol Sci. 2005 Jan 29;360(1453):5–12. doi: 10.1098/rstb.2004.1574 15713585 PMC1636108

[4] Galgani F, Claro F, Depledge M, Fossi C. Monitoring the impact of litter in large vertebrates in the Mediterranean Sea within the European Marine Strategy Framework Directive (MSFD): Constraints, specificities and recommendations. Mar Environ Res. 2014 Sep;100:3–9. doi: 10.1016/j.marenvres.2014.02.003 24612883

[5] Laist DW. Impacts of Marine Debris: Entanglement of Marine Life in Marine Debris Including a Comprehensive List of Species with Entanglement and Ingestion Records. In: Coe JM, Rogers DB, editors. Marine Debris [Internet]. Springer New York; 1997 [cited 2016 Feb 18]. p. 99–139. (Springer Series on Environmental Management). Available from: http://link.springer.com/chapter/10.1007/978-1-4613-8486-1_10

[6] Wright SL, Thompson RC, Galloway TS. The physical impacts of microplastics on marine organisms: A review. Environ Pollut. 2013 Jul;178:483–92. doi: 10.1016/j.envpol.2013.02.031 23545014

[7] Tanabe S, Iwata H, Tatsukawa R. Global contamination by persistent organochlorines and their ecotoxicological impact on marine mammals. Sci Total Environ. 1994 Sep 16;154(2–3):163–77. doi: 10.1016/0048-9697(94)90086-8 7973605

[8] IPCC. Climate Change 2014: Synthesis Report. Contribution of Working Groups I, II and III to the Fifth Assessment Report of the Intergovernmental Panel on Climate Change [Core Writing Team, R.K. Pachauri and L.A. Meyer (eds.)]. Geneva, Switzerland: IPCC; 2014.

[9] IPCC. Summary for policymakers. In: Climate Change 2014: Impacts, Adaptation, and Vulnerability Part A: Global and Sectoral Aspects Contribution of Working Group II to the Fifth Assessment Report of the Intergovernmental Panel on Climate Change. Cambridge, United Kingdom and New York, NY, USA,: Cambridge University Press; 2014. p. 1–32.

[10] United Nations. Transforming Our World: The 2030 Agenda for Sustainable Development [Internet]. New York: UN Publishing; 2015 [cited 2021 Jan 11]. Report No.: A/RES/70/1. Available from: https://sdgs.un.org/sites/default/files/publications/21252030%20Agenda%20for%20Sustainable%20Development%20web.pdf

[11] Costanza R. The ecological, economic, and social importance of the oceans. Ecol Econ. 1999 Nov;31(2):199–213.

[12] Pieraccini M, Novitz T. Legal perspectives on sustainability. Policy Press; 2020.

[13] Chen CL, Tsai CH. Marine environmental awareness among university students in Taiwan: a potential signal for sustainability of the oceans. Environ Educ Res. 2016 Oct 2;22(7):958–77.

[14] Guest H, Lotze HK, Wallace D. Youth and the sea: Ocean literacy in Nova Scotia, Canada. Mar Policy. 2015 Aug;58:98–107.

[15] Kollmuss A, Agyeman J. Mind the Gap: Why do people act environmentally and what are the barriers to pro-environmental behavior? Environ Educ Res. 2002 Aug 1;8(3):239–60.

[16] Schild R. Environmental citizenship: What can political theory contribute to environmental education practice? J Environ Educ. 2016 Jan 2;47(1):19–34.

[17] McKinley E, Fletcher S. Improving marine environmental health through marine citizenship: A call for debate. Mar Policy. 2012 May;36(3):839–43.

[18] Gelcich S, Buckley P, Pinnegar JK, Chilvers J, Lorenzoni I, Terry G, et al. Public awareness, concerns, and priorities about anthropogenic impacts on marine environments. Proc Natl Acad Sci. 2014 Oct 21;111(42):15042–7. doi: 10.1073/pnas.1417344111 25288740 PMC4210304

[19] Pearson E, Mellish S, Sanders B, Litchfield C. Marine wildlife entanglement: Assessing knowledge, attitudes, and relevant behaviour in the Australian community. Mar Pollut Bull. 2014 Dec 15;89(1–2):136–48. doi: 10.1016/j.marpolbul.2014.10.014 25455820

[20] Barney EC, Mintzes JJ, Yen CF. Assessing Knowledge, Attitudes, and Behavior Toward Charismatic Megafauna: The Case of Dolphins. J Environ Educ. 2005 Jan 1;36(2):41–55.

[21] Blake J. Overcoming the ‘value‐action gap’ in environmental policy: Tensions between national policy and local experience. Local Environ. 1999 Oct 1;4(3):257–78.

[22] Owens S. ‘Engaging the public’: information and deliberation in environmental policy. Environ Plan A. 2000;32(7):1141–8.

[23] Barr S. Strategies for sustainability: citizens and responsible environmental behaviour. Area. 2003 Sep 1;35(3):227–40.

[24] Estrada M, Schultz PW, Silva-Send N, Boudrias MA. The Role of Social Influences on Pro-Environment Behaviors in the San Diego Region. J Urban Health. 2017 Apr 1;94(2):170–9. doi: 10.1007/s11524-017-0139-0 28265806 PMC5391335

[25] Hamilton LC, Safford TG. Environmental Views from the Coast: Public Concern about Local to Global Marine Issues. Soc Nat Resour. 2015 Jan 2;28(1):57–74.

[26] Faulks K. Citizenship. Psychology Press; 2000. 212 p.

[27] Fletcher S, Potts J. Ocean Citizenship: An Emergent Geographical Concept. Coast Manag. 2007 Sep 4;35(4):511–24.

[28] McKinley E. A Critical evaluation of the application of marine citizenship in sustainable marine management in the UK [Internet] [Doctorate Thesis (Doctorate)]. Bournemouth University; 2010. Available from: http://eprints.bournemouth.ac.uk/18830/

[29] McKinley E, Fletcher S. Individual responsibility for the oceans? An evaluation of marine citizenship by UK marine practitioners. Ocean Coast Manag. 2010 Jul;53(7):379–84.

[30] Rees S, Fletcher S, Glegg G, Marshall C, Rodwell L, Jefferson R, et al. Priority questions to shape the marine and coastal policy research agenda in the United Kingdom. Mar Policy. 2013 Mar;38:531–7.

[31] Stoll-Kleemann S. Feasible Options for Behavior Change Toward More Effective Ocean Literacy: A Systematic Review. Front Mar Sci. 2019 May 28;6:UNSP 273.

[32] Marshall TH. Citizenship and social class. Vol. 11. CUP Cambridge; 1950.

[33] Dobson A. Citizenship and the Environment. OUP Oxford; 2003. 244 p.

[34] Rio Declaration on Environment and Development [Internet]. A/CONF.151/26 1992. Available from: https://www.un.org/en/development/desa/population/migration/generalassembly/docs/globalcompact/A_CONF.151_26_Vol.I_Declaration.pdf

[35] United Nations Economic Commission for Europe. Convention on Access to Information, Public Participation in Decision-making and Access to Justice in Environmental Matters. Jun 25, 1998.

[36] Holder J. Environmental Assessment: The Regulation of Decision Making. Oxford, New York: Oxford University Press; 2006. 400 p.

[37] Lee M. Access to Justice at EU Level in Environmental Law [Internet]. Rochester, NY: Social Science Research Network; 2012 May [cited 2016 Nov 15]. Report No.: ID 2062252. Available from: https://papers.ssrn.com/abstract=2062252

[38] Lee M, Abbot C. The Usual Suspects? Public Participation Under the Aarhus Convention. Mod Law Rev. 2003 Jan 1;66(1):80–108.

[39] Nadal C. Pursuing Substantive Environmental Justice: The Aarhus Convention as a ‘Pillar’of Empowerment. Environ Law Rev. 2008;10(1):28–45.

[40] Peters B. Towards the Europeanization of Participation? Reflecting on the Functions and Beneficiaries of Participatory Rights in EU Environmental Law. In: Fraenkel-Haeberle C, Kropp S, Palermo F, Sommermann KP, editors. Citizen Participation in Multi-level Democracies [Internet]. Leiden: Koninklijke Brill NV; 2015 [cited 2016 Nov 15]. p. 311–33. Available from: https://papers.ssrn.com/abstract=2422378

[41] Participation Steele J. and Deliberation in Environmental Law: Exploring a Problem‐solving Approach. Oxf J Leg Stud. 2001 Jan 9;21(3):415–42.

[42] Robottom I, Hart P. Behaviorist EE research: Environmentalism as individualism. J Environ Educ. 1995;26(2):5–9.

[43] Johnson RB, Onwuegbuzie AJ, Turner LA. Toward a Definition of Mixed Methods Research. J Mix Methods Res. 2007 Jan 4;1(2):112–33.

[44] Creswell JW. Research design: Qualitative, quantitative, and mixed methods approaches [Internet]. Fourth. Sage publications; 2014 [cited 2015 Nov 4]. Available from: http://books.google.com/books?hl=en&lr=&id=EbogAQAAQBAJ&oi=fnd&pg=PR1&dq=info:wZfwqvnlv2gJ:scholar.google.com&ots=caeQuULzx7&sig=8YZqBiF7-OpNZL2n9ypaUPG8BKk

[45] Marcus GE. Ethnography in/of the World System: The Emergence of Multi-Sited Ethnography. Annu Rev Anthropol. 1995;24(1):95–117.

[46] Saldaña J. The coding manual for qualitative researchers. SAGE Publications Ltd; 2016.

[47] UK Political Info. Voter turnout at UK general elections 1945–2017 | UK Political Info [Internet]. UK Political Info. 2019 [cited 2019 Jun 18]. Available from: http://www.ukpolitical.info/Turnout45.htm

[48] Buchan PM. Investigating marine citizenship and its role in creating good marine environmental health [Internet]. University of Exeter; 2021 [cited 2021 Jun 21]. Available from: http://hdl.handle.net/10871/126112

[49] Marine and Coastal Access Act [Internet]. c. 23 2009. Available from: http://www.legislation.gov.uk/ukpga/2009/23/contents

[50] Arnstein SR. A Ladder Of Citizen Participation. J Am Inst Plann. 1969 Jul 1;35(4):216–24.

[51] Black J. Proceduralizing regulation: part I. Oxf J Leg Stud. 2000;20(4):597–614.

[52] Pieraccini M. Rethinking Participation in Environmental Decision-Making: Epistemologies of Marine Conservation in South-East England. J Environ Law. 2015 Jan 3;27(1):45–67.

[53] Parsons ECM, Favaro B, Aguirre AA, Bauer AL, Blight LK, Cigliano JA, et al. Seventy-One Important Questions for the Conservation of Marine Biodiversity. Conserv Biol. 2014 Oct 1;28(5):1206–14. doi: 10.1111/cobi.12303 24779474 PMC4264944

[54] Potts T O’Higgins T, Hastings E. Oceans of opportunity or rough seas? What does the future hold for developments in European marine policy? Philos Trans R Soc Lond Math Phys Eng Sci. 2012 Dec 13;370(1980):5682–700. doi: 10.1098/rsta.2012.0394 23129717

[55] Heck N, Paytan A, Potts DC, Haddad B. Coastal residents’ literacy about seawater desalination and its impacts on marine ecosystems in California. Mar Policy. 2016 Jun;68:178–86.

[56] Hawthorne M, Alabaster T. Citizen 2000: development of a model of environmental citizenship. Glob Environ Change. 1999 Apr;9(1):25–43.

[57] Freire P. Pedagogy of the oppressed. Harmondsworth: Penguin; 1972.

[58] Martin I. Adult education, lifelong learning and citizenship: some ifs and buts. Int J Lifelong Educ. 2003 Nov 1;22(6):566–79.

[59] Hoskins BL, Mascherini M. Measuring Active Citizenship through the Development of a Composite Indicator. Soc Indic Res. 2009 Feb 1;90(3):459–88.

[60] Jefferson RL, Bailey I, Laffoley D d′A., Richards JP, Attrill MJ. Public perceptions of the UK marine environment. Mar Policy. 2014 Jan;43:327–37.

[61] Bird EAR. The Social Construction of Nature: Theoretical Approaches to the History of Environmental Problems. Environ Rev ER. 1987;11(4):255–64.

[62] Bonter DN, Cooper CB. Data validation in citizen science: a case study from Project FeederWatch. Front Ecol Environ. 2012;10(6):305–7.

[63] Garcia-Soto C, van der Meeren G, Busch J, Delany J, Domegan C, Dubsky K, et al. Advancing Citizen Science for Coastal and Ocean Research. Position Paper 23 of the European Marine Board, Ostend, Belgium; 2017. 112 p.

[64] Communication Foxwell-Norton K., culture, community and country: the lost seas of environmental policy. Continuum. 2013;27(2):267–82.

[65] Lazarus R. Super Wicked Problems and Climate Change: Restraining the Present to Liberate the Future. Georget Law Fac Publ Works [Internet]. 2009 Jan 1; Available from: https://scholarship.law.georgetown.edu/facpub/159

[66] Jelin E. Towards a Global Environmental Citizenship? Citizsh Stud. 2000 Feb 1;4(1):47–63.

[67] Jabar MA, Jr CQR, Collado ZC. Exploring marine citizenship among young people in select urban and rural villages in the Philippines. Appl Environ Educ Commun. 2018 Jan 2;17(1):67–78.

[68] Stech R. Costs barriers to environmental judicial review; A study in environmental justice [Internet]. Cardiff University; 2013 [cited 2022 Apr 6]. Available from: https://orca.cardiff.ac.uk/47605/1/PhD_Radoslaw_Stech_0538599_final.pdf

[69] United Nations Economic Commission for Europe. Convention on Environmental Impact Assessment in a Transboundary Context [Internet]. 1991. Available from: https://unece.org/environment-policyenvironmental-assessment/introduction

[70] Lorenzoni I, Nicholson-Cole S, Whitmarsh L. Barriers perceived to engaging with climate change among the UK public and their policy implications. Glob Environ Change. 2007 Aug;17(3–4):445–59.

[71] Sundeen RA, Raskoff SA, Garcia MC. Differences in perceived barriers to volunteering to formal organizations: Lack of time versus lack of interest. Nonprofit Manag Leadersh. 2007 Mar 1;17(3):279–300.

[72] Chawla L. Life Paths Into Effective Environmental Action. J Environ Educ. 1999 Jan 1;31(1):15–26.

[73] Chawla L, Cushing DF. Education for strategic environmental behavior. Environ Educ Res. 2007 Sep 1;13(4):437–52.

[74] Diani M. The concept of social movement. Sociol Rev. 1992;40(1):1–25.

[75] Tindall DB. Social networks, identification and participation in an environmental movement: Low-medium cost activism within the British Columbia Wilderness Preservation Movement. Can Rev Sociol Anthropol-Rev Can Sociol Anthropol. 2002 Nov;39(4):413–52.

[76] Wright KB. Researching Internet-Based Populations: Advantages and Disadvantages of Online Survey Research, Online Questionnaire Authoring Software Packages, and Web Survey Services. J Comput-Mediat Commun. 2005 Apr 1;10(3):JCMC1034.

