# Supplementary material for: Marine citizenship: The right to participate in the transformation of the human-ocean relationship for sustainability
Source: PLoS One. 2023 Mar 13;18(3):e0280518. doi: 10.1371/journal.pone.0280518 (PMC10010517; doi:10.1371/journal.pone.0280518)
Supplement: S1 File — Online and print survey used in this study to investigate marine citizenship and a range of factors influencing it, in a population of marine citizens. (PDF) [file pone.0280518.s001.pdf]

Marine Citizenship [CoCoasts]

Page 1: Introduction and consent

Thank you for clicking to participate in this survey.

About this research

My name is Pamela Buchan and I am a post-graduate researcher at Exeter University. My PhD research investigates marine citizenship - people voluntarily performing activities for the common good of the marine environment - and how it can help promote a healthy marine environment. It looks at a number of UK case studies, of which Capturing our Coasts forms one, which differ in the way people engage with marine environmental issues. The aim of the research is to better understand the different factors related to citizenship and how these influence citizenship, specifically marine. This research has been approved by the University of Exeter Ethics Committee.

How will data be collected and used?

Data will be collected through this survey, interviews, focus group, and shadowing of participants at events. I will ensure participants are fully informed and consent to participation. Participants have the right to withdraw from the research should they change their mind up until the point at which data has been anonymised, after which it will be unidentifiable. Data collected will include answers provided to survey questions, audio recordings of interviews, and notes taken by myself. It will be used to produce a PhD thesis and may be published in

scientific journals. To protect the privacy of participants, your name will not be associated with any information you provide. Data will be safely stored at the university and destroyed five years after the research has been completed.

The Survey

This survey includes a range of questions designed to investigate your opinions and values about citizenship of all kinds, with particular emphasis on your marine citizenship activities. It will also collect general demographic information. In addition to closed questions, there are also opportunities for you to use your own words for some questions. With the exception of consent, questions are optional or have a ‘prefer not to answer’ option, but the more fully you are able to answer the survey the more informative the research will be. Remember that the data is anonymised so answers will not be identifiable to you personally unless you additionally volunteer and are asked to participate in a focus group. The survey should take around 30 minutes to complete, depending on how much detail you choose to provide, and is best displayed on a computer screen rather than on a mobile phone. You can save the survey and return to it at a later time but may find it easier to complete it in one sitting. At the end of the survey you will be given the option to volunteer to participate in a focus group. Expression of interest neither guarantees nor commits you to participate in the interview or focus group. You can print out your responses at the end, should you wish to.

Questions

If you have any questions about this research either during or after participation or wish to withdraw from the research after having participated, please do not hesitate to contact me. You can reach me on email at pb381@exeter.ac.uk. Please make a note of my email address for future reference.

Prize draw

Survey respondents can choose to be entered into a prize draw for a £25 Amazon voucher. To opt into this draw, please provide an email address when requested to do so at the end of the survey.

Consent

1. I have read the information provided and understand the aims and objectives of this research. I understand that data collected in this research will be used for publication in a PhD thesis and scientific journals, that my data will not be connected to my name and that any data which might be personally identifiable will only be used with my additional consent for that data. I understand that I can stop participating at any time without prejudice and that I can ask to remove my data from the study prior to it being anonymised after which point it won't be possible

to identify my data. ✱ *Required*

- ○ Yes

2. I agree to participate in this survey. ✱ *Required*

- ○ Yes

3. I confirm that I am 18 years old or over. ✱ *Required*

- ○ Yes

Page 2: Some general information about you

4. Which gender do you identify as?

5. Please type your age in years in the box.

Please enter a whole number (integer).

6. If you have an educational or professional background related to the environmental or specifically marine issues, please specify. e.g. I have a degree in marine science; I have worked for a conservation NGO.

7. For how long have you lived in the local area where you live now? Please type in the number of years. If less than a year, type in 0.

Please enter a whole number (integer).

8. Please provide the first part of your home postcode, e.g. PL1. This is to determine how far you live from the sea.

Please enter a valid UK postcode.

9. How often do you visit the sea? Please select the closest option.

- Every day
- Once a week
- Once a month
- A few times a year
- Less often

Page 3: Thinking about citizenship

10. You are participating in this survey because of your citizenship involvement with Capturing our Coasts. What motivates you to be involved in this and other citizenship activities related to the marine environment?

10.a. In what ways has your participation in Capturing our Coasts influenced your marine and other citizenship participation?

11. In which of the following ways do you actively participate generally as a citizen? Please select all that apply.

- ☐ Vote in elections
- ☐ Join in with campaigns organised by others on issues that are important to you
- ☐ Start up your own campaigns on issues that are important to you

- [ ] Sign petitions
- [ ] Volunteer with local organisations doing work in your local area
- [ ] Volunteer with organisations working nationally
- [ ] Volunteer with organisations working internationally
- [ ] Pick up litter
- [ ] Participate in environmental action e.g. nature monitoring, citizen science, land regeneration
- [ ] Member of a trade union

12. In which of the following ways do you actively participate specifically as a marine citizen? Please select all that apply.

- [ ] Pick up beach litter
- [ ] Don't drop litter on the beach
- [ ] Stick to recognised paths to avoid erosion
- [ ] Support marine conservation charities or campaigns e.g. donations of time or money, campaign activities
- [ ] Organise marine environmental action e.g. beach cleans, public events
- [ ] Make consumer choices to protect the environment e.g. certified sustainble fish, toiletries without plastic microbeads
- [ ] Make lifestyle choices to directly protect the marine environment e.g. not consume fish
- [ ] Make lifestyle choices to indirectly protect the marine environment e.g. reducing your carbon footprint by using less fossil fuel based energy, paying more for green energy
- [ ] Make conscientious choices in your recreational pursuits e.g. not finning close to sea bed when diving, choosing less harmful antifoulant paints for your boat, ensuring all fishing gear is retrieved
- [ ] Participate in marine citizen science

12.a. Are there other ways in which you feel you act as a marine citizen?

12.b. What things enable you to be an active marine citizen?

12.c. What things prevent or get in the way of your marine citizenship activities?

13. In what ways do you think marine citizenship is important for marine environmental health?

14. Please rank on the following scale how active a marine citizen you believe you are.

Please don't select more than 1 answer(s) per row.

	1 - I never consider the impact I have on the marine environment	2	3	4	5	6	7 - I always consider the impact my actions have on the marine environment
As a marine citizen	☐	☐	☐	☐	☐	☐	☐

Page 4: Marine policy and decision-making

15. Are you aware of any international/EU/national legislation which promotes the processes of citizenship and public participation in environmental and marine decision-making? Please describe what you are aware of?

16. To what extent do you think each of the following are involved in marine and coastal decision-making?

Please don't select more than 1 answer(s) per row.

	1 – Not at all involved	2	3	4	5 – Strongly involved	Don’t know
Local residents	☐	☐	☐	☐	☐	☐
Local government/local politicians	☐	☐	☐	☐	☐	☐
National Government/Government ministers	☐	☐	☐	☐	☐	☐
International Government/Government ministers	☐	☐	☐	☐	☐	☐
Environmental organisations	☐	☐	☐	☐	☐	☐
Aquatic recreation organisations	☐	☐	☐	☐	☐	☐

17. In what ways do you think marine citizenship is important for the process of marine decision-making?

18. Have you ever been involved in a **marine** decision-making activity? E.g. marine conservation zone consultation, submission to a government enquiry

- Yes
- No

18.a. If yes, please list any such activities and how you contributed.

18.b. Please describe in what ways and to what extent you think your views were or were not taken into account?

19. If you are not happy with an environmental decision from a regulatory body and/or law or regulation, which avenues are available to you for legal redress?
Please also describe if you have ever used one, or how else you can raise your concerns. If you don't know, please state so.

Page 5: You and the sea

20. To what extent do you feel a weak or a strong sense of belonging to the **built and natural features** of the following environments?

Please don't select more than 1 answer(s) per row.

	1 – No sense of belonging	2	3	4	5 – Very strong sense of belonging	Don’t know
The neighbourhood where you live	☐	☐	☐	☐	☐	☐
The region of the country where you live	☐	☐	☐	☐	☐	☐
The UK	☐	☐	☐	☐	☐	☐
Europe	☐	☐	☐	☐	☐	☐
The Earth / The whole world	☐	☐	☐	☐	☐	☐

21. To what extent do you feel a weak or a strong sense of belonging to the **people and society** in the following areas?

Please don't select more than 1 answer(s) per row.

	1 – No sense of belonging	2	3	4	5 – Very strong sense of belonging	Don’t know
The neighbourhood where you live	☐	☐	☐	☐	☐	☐
The region of the country where you live	☐	☐	☐	☐	☐	☐
The UK	☐	☐	☐	☐	☐	☐
Europe	☐	☐	☐	☐	☐	☐

The Earth / The whole world	☐	☐	☐	☐	☐	☐

22. How attached are you to your local beach or coastline? Please rate how much you agree or disagree with each statement.

Please don't select more than 1 answer(s) per row.

	1 - Strongly disagree	2	3	4	5 - Strongly agree	Don't know	Not applicable, I don't live near a beach or coastline
Living in this place was my conscious choice.	☐	☐	☐	☐	☐	☐	☐
I have strong family connections to this place.	☐	☐	☐	☐	☐	☐	☐
The marine environment is the best place for the things I like to do.	☐	☐	☐	☐	☐	☐	☐
There are many coasts in the UK and in the world where I could live.	☐	☐	☐	☐	☐	☐	☐

23. How do you feel about the marine and coastal environment in general? Please rate how much you agree or disagree with each statement.

Please don't select more than 1 answer(s) per row.

	1 - Strongly disagree	2	3	4	5 - Strongly agree	Don’t know	Not applicable
I miss the sea when I am not there	☐	☐	☐	☐	☐	☐	☐
I am proud of the marine environment	☐	☐	☐	☐	☐	☐	☐
The sea is part of me	☐	☐	☐	☐	☐	☐	☐
I want to be engaged in affairs of the sea	☐	☐	☐	☐	☐	☐	☐
I take pleasure in looking at the sea	☐	☐	☐	☐	☐	☐	☐

24. In what way do you feel dependent upon the marine environment?

Please don't select more than 1 answer(s) per row.

	1 - Strongly disagree	2	3	4	5 - Strongly agree	Don’t know
I depend upon the sea for my livelihood	☐	☐	☐	☐	☐	☐
I depend upon the sea for my wellbeing	☐	☐	☐	☐	☐	☐
I depend upon the sea for my recreation or other interests	☐	☐	☐	☐	☐	☐

Page 6: A bit more about you and your citizenship

25. The following statements are about the world around you and yourself. Think about each statement and indicate to what extent you agree or disagree with them. *[If you have difficulty viewing the whole table, you can select the option to show it as seperate questions.]*

Please don't select more than 1 answer(s) per row.

	1 - Strongly disagree	2	3	4	5 - Strongly agree	Don’t know
The balance of nature is very delicate and easily upset.	☐	☐	☐	☐	☐	☐
When humans interfere with nature it often produces disastrous consequences.	☐	☐	☐	☐	☐	☐
Humans are severely abusing the environment.	☐	☐	☐	☐	☐	☐
If things continue on their present course, we will soon experience a major ecological catastrophe.	☐	☐	☐	☐	☐	☐
I admire people who own expensive homes, cars and clothes.	☐	☐	☐	☐	☐	☐
Some of the most important achievements in life include possessing things such as expensive cars or clothes.	☐	☐	☐	☐	☐	☐
I don't place much emphasis on the amount of material objects people own as a sign of their success.	☐	☐	☐	☐	☐	☐

I worry a great deal about climate change.	☐	☐	☐	☐	☐	☐
Climate change is a more serious challenge than our politicians like to think.	☐	☐	☐	☐	☐	☐
I spend a lot of time in natural settings (woods, hills, countryside, lakes, ocean).	☐	☐	☐	☐	☐	☐
Being a part of an ecosystem is an important part of who I am.	☐	☐	☐	☐	☐	☐
I have a lot in common with environmentalists as a group.	☐	☐	☐	☐	☐	☐
Behaving responsibly toward the Earth - living a sustainable lifestyle - is part of my moral code.	☐	☐	☐	☐	☐	☐
I feel that I receive spiritual sustenance from experiences with nature.	☐	☐	☐	☐	☐	☐

26. Below some people are briefly described. Please read each description and say how much each person is or is not like you. Please complete every line. *[If you have difficulty viewing the whole table, you can select the option to show it as seperate questions.]*

Please don't select more than 1 answer(s) per row.

Please select at least 21 answer(s).

	Very much like me	Like me	Somewhat like me	A little like me	Not like me	Not like me at all	Don't know

Thinking up new ideas and being creative is important to them. They like to do things in their own original way.	☐	☐	☐	☐	☐	☐	☐
It is important to them to be rich. They want to have a lot of money and expensive things.	☐	☐	☐	☐	☐	☐	☐
They thinks it is important that every person in the world should be treated equally. They believe everyone should have equal opportunities in life.	☐	☐	☐	☐	☐	☐	☐
It's important to them to show their abilities. They want people to admire what they do.	☐	☐	☐	☐	☐	☐	☐

It is important to them to live in secure surroundings. They avoid anything that might endanger their safety.	☐	☐	☐	☐	☐	☐	☐
They like surprises and are always looking for new things to do. They think it is important to do lots of different things in life.	☐	☐	☐	☐	☐	☐	☐
They believe that people should do what they're told. They think people should follow rules at all times, even when no-one is watching.	☐	☐	☐	☐	☐	☐	☐

It is important to them to listen to people who are different from them. Even when they disagree with people, they still want to understand those people.	☐	☐	☐	☐	☐	☐	☐
It is important to them to be humble and modest. They try not to draw attention to themselves.	☐	☐	☐	☐	☐	☐	☐
Having a good time is important to them. They like to “spoil” themself.	☐	☐	☐	☐	☐	☐	☐
It is important to them to make their own decisions about what they do. They like to be free and not depend on others.	☐	☐	☐	☐	☐	☐	☐

It's very important to them to help the people around them. They want to care for others' well-being.	☐	☐	☐	☐	☐	☐	☐
Being very successful is important to them. They hope people will recognise their achievements.	☐	☐	☐	☐	☐	☐	☐
It is important to them that the government ensures their safety against all threats. They want the state to be strong so it can defend its citizens.	☐	☐	☐	☐	☐	☐	☐
They look for adventures and like to take risks. They want to have an exciting life.	☐	☐	☐	☐	☐	☐	☐

It is important to them always to behave properly. They want to avoid doing anything people would say is wrong.	☐	☐	☐	☐	☐	☐	☐
It is important to them to get respect from others. They want people to do what they say.	☐	☐	☐	☐	☐	☐	☐
It is important to them to be loyal to their friends. They want to devote themselves to people close to them.	☐	☐	☐	☐	☐	☐	☐
They strongly believe that people should care for nature. Looking after the environment is important to them.	☐	☐	☐	☐	☐	☐	☐

Tradition is important to them. They try to follow the customs handed down by their religion or their family.	☐	☐	☐	☐	☐	☐	☐
They seek every chance they can to have fun. It is important to them to do things that give them pleasure.	☐	☐	☐	☐	☐	☐	☐

Page 7: And finally...

27. Which of the following is the highest level of educational qualification you hold?

- O Level / GCSE / NVQ Level 1-2 or equivalent
- A Level, AS/A2 Level, NVQ Level 3-4 or equivalent
- Undergraduate degree (e.g. BA, BSc)
- Postgraduate degree (e.g. MA, MSc, PhD)
- None of the above
- Prefer not to answer

28. Gross household income combines your income with that of your partner or any other household members with whom you share financial responsibilities before any taxes or deductions. What is your gross HOUSEHOLD income from any source?

- Under £5,000 per year
- £5,000 to £9,999 per year
- £10,000 to £14,999 per year
- £15,000 to £19,999 per year
- £20,000 to £29,999 per year
- £30,000 to £39,999 per year
- £40,000 to £49,999 per year
- £50,000 to £99,999 per year
- £100,000 and over
- Don't know
- Prefer not to answer

29. Which party do you most closely align with politically?

- Conservative
- Labour

- Liberal Democrat
- Scottish National Party (SNP)/ Plaid Cymru
- Green Party
- Don't know
- Would not vote
- Prefer not to answer
- Other party (please specify)

29.a. If you selected Other, please specify:

To fully understand the findings of the survey and the way individuals pursue marine citizenship activities, I will be working with a small number of participants in activities such as focus groups, interviews, and observing at events. If you would be interested in further participation of this nature, please provide your name and a contact email address/telephone number below. If selected I will contact you to discuss the next stage.

30. Name:

31. Email address:

32. If you would like to be entered into the prize draw for a £25 Amazon voucher, please provide your email address below. This data will be removed from the rest of the survey so please complete this box even if you have already provided contact details.

33. If you have comments, please use this space to share them.

Page 8: Thank you!

Thank you for participating in this survey. The answers you have provided will help scientists to better understand the relationships we have with the sea and what drives us to want to improve its health.

As a reminder, your data is confidential and you will not be personally identifiable from it. Should you have any questions about this survey, further opportunities to participate, or generally about this research, please do get in touch: pb381@exeter.ac.uk.
